# Common electronic origin of superconductivity in (Li,Fe)OHFeSe bulk superconductor and single-layer FeSe/SrTiO_3_ films

**DOI:** 10.1038/ncomms10608

**Published:** 2016-02-08

**Authors:** Lin Zhao, Aiji Liang, Dongna Yuan, Yong Hu, Defa Liu, Jianwei Huang, Shaolong He, Bing Shen, Yu Xu, Xu Liu, Li Yu, Guodong Liu, Huaxue Zhou, Yulong Huang, Xiaoli Dong, Fang Zhou, Kai Liu, Zhongyi Lu, Zhongxian Zhao, Chuangtian Chen, Zuyan Xu, X. J. Zhou

**Affiliations:** 1 National Lab for Superconductivity, Beijing National Laboratory for Condensed Matter Physics, Institute of Physics, Chinese Academy of Sciences, Beijing 100190, China; 2 Department of Physics and Beijing Key Laboratory of Opto-electronic Functional Materials and Micro-nano Devices, Renmin University of China, Beijing 100872, China; 3 Collaborative Innovation Center of Quantum Matter, Beijing 100871, China; 4 Technical Institute of Physics and Chemistry, Chinese Academy of Sciences, Beijing 100190, China

## Abstract

The mechanism of high-temperature superconductivity in the iron-based superconductors remains an outstanding issue in condensed matter physics. The electronic structure plays an essential role in dictating superconductivity. Recent revelation of distinct electronic structure and high-temperature superconductivity in the single-layer FeSe/SrTiO_3_ films provides key information on the role of Fermi surface topology and interface in inducing or enhancing superconductivity. Here we report high-resolution angle-resolved photoemission measurements on the electronic structure and superconducting gap of an FeSe-based superconductor, (Li_0.84_Fe_0.16_)OHFe_0.98_Se, with a *T*_c_ at 41 K. We find that this single-phase bulk superconductor shows remarkably similar electronic behaviours to that of the superconducting single-layer FeSe/SrTiO_3_ films in terms of Fermi surface topology, band structure and the gap symmetry. These observations provide new insights in understanding high-temperature superconductivity in the single-layer FeSe/SrTiO_3_ films and the mechanism of superconductivity in the bulk iron-based superconductors.

## Abstract (web summary)

The mechanism of high-temperature superconductivity in the iron-based materials remains not fully understood. Here, the authors report on ARPES measurements on an FeSe-based bulk superconductor, whose electronic properties are found to be similar to those of single-layer FeSe/STO films.

## Graphical abstract

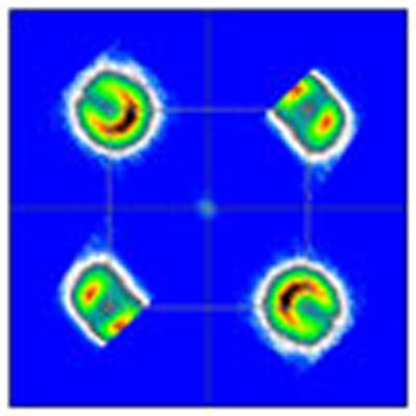

Iron-based superconductors^1^,^2^,^3^ represent the second class of high-temperature superconductors after the first discovery of high-temperature superconductivity in cuprates^4^. The electronic structure of the iron-based compounds usually consists of hole-like bands near the Brillouin zone centre and electron-like bands near the Brillouin zone corners, leading to proposals that the electron scattering between the hole pockets around the Brillouin zone centre and the electron pockets around the Brillouin zone corners is a viable mechanism for electron pairing in the iron-based superconductors^5^,^6^,^7^,^8^,^9^,^10^,^11^. With the discovery of the A_*x*_Fe_2−*y*_Se_2_ (A=K, Cs, Rb, Tl and so on) superconductors^3^,^12^,^13^,^14^, such a Fermi surface nesting picture is challenged, because the superconducting phase in A_*x*_Fe_2−*y*_Se_2_ superconductors does not contain hole pockets near the Brillouin zone centre^3^,^14^,^15^,^16^,^17^,^18^. The latest discovery of possible high-temperature superconductivity above 65 K in the single-layer FeSe film grown on the SrTiO_3_ substrate (denoted as FeSe/SrTiO_3_ film hereafter) puts even stronger constraints on the Fermi surface nesting picture, because there is no Fermi surface present near the zone centre at all^19^,^20^,^21^,^22^,^23^. Although the implications of the Fermi surface topology in the A_*x*_Fe_2−*y*_Se_2_ superconductors and superconducting single-layer FeSe/SrTiO_3_ films are significant, the material-specific complications make the conclusion on the role of Fermi surface topology ambiguous. The A_*x*_Fe_2−*y*_Se_2_ superconductors are well-known to have problems of inhomogeneity, phase separation and small superconducting volume fraction. The exact nature of the superconducting phase remains unclear and its coexistence with the insulating phase suggests that interface may play an important role in the superconductivity^24^,^25^,^26^,^27^. There are strong indications that interface in the single-layer FeSe/SrTiO_3_ films plays an important role in giving rise to high-temperature superconductivity^19^,^20^,^21^,^22^,^23^,^28^,^29^,^30^,^31^,^32^,^33^,^34^. With the complications of the interface effect and phase separation problem involved in A_*x*_Fe_2−*y*_Se_2_ superconductors and superconducting single-layer FeSe/SrTiO_3_ films, it becomes less straightforward to conclude whether the same effect of Fermi surface topology on superconductivity is operative in all the iron-based superconductors.

In this study, we report high-resolution angle-resolved photoemission (ARPES) measurements on a new FeSe-based superconductor, (Li_1−*x*_Fe_*x*_)OHFe_1−*y*_Se, with a superconducting critical temperature at 41 K (refs 35, 36, 37, 38, 39, 40). We find that this superconductor contains electron pocket(s) near the Brillouin zone conners without hole pockets near the zone centre. The superconducting gap around the electron-like Fermi surface near the zone corner is nearly isotropic without nodes and its temperature dependence follows the usual Bardeen-Cooper-Schrieffer form. These behaviours are strikingly similar to that of the superconducting single-layer FeSe/SrTiO_3_ films. This is a single-phase bulk superconductor with a relatively high *T*_c_*∼*41 K, free from the complication of phase separation and interface effect, which shows electron-like Fermi pockets only. Our observations provide insights on the superconductivity mechanism in the iron-based bulk superconductors and the origin of high-temperature superconductivity in the single-layer FeSe/SrTiO_3_ films.

## Results

### Fermi surface and band structure

**Figure 1:**
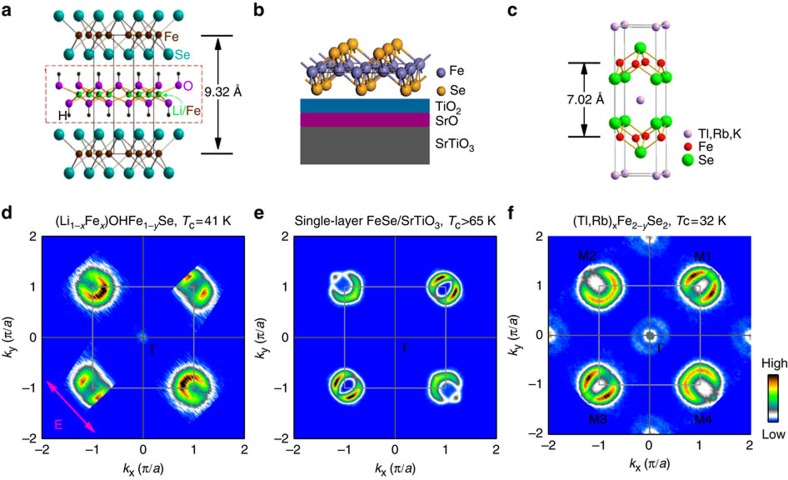
Crystal structure and Fermi surface of three FeSe-based superconductors. (**a**) The crystal structure of (Li_1−*x*_Fe_*x*_)OHFe_1−*y*_Se. The *c* lattice constant is 9.318 Å for optimally doped sample with a *T*_c_ at 42 K (refs 36, 39). (**b**) Schematic structure of single-layer FeSe film deposited on a SrTiO_3_ substrate^19^. The thickness of the FeSe layer is ∼5.5 Å. (**c**) Schematic structure of (Tl,Rb)_*x*_Fe_2−*y*_Se_2_ (ref. 12). The distance of the adjacent FeSe layers is ∼7.02 Å. (**d**) Fermi surface mapping of (Li_0.84_Fe_0.16_)OHFe_0.98_Se superconductor measured at 20 K. The spectral weight distribution was obtained by integrating the measured photoemission spectra (EDCs) within [−10,+10] meV energy window with respect to the Fermi level as a function of *k*_x_ and *k*_y_. (**e**,**f**) Fermi surface of a superconducting single-layer FeSe/SrTiO_3_ film^20^ and (Tl,Rb)_*x*_Fe_2−*y*_Se_2_ (ref. 17). For convenience, the four equivalent M points are labelled as M1(*π*,*π*), M2(−*π*,*π*), M3(−*π*,−*π*) and M4(*π*,−*π*). The light-grey arrow in **d** marks the main electric field direction on the sample surface from the light source. In **d**–**f**, the data around M1 and M4 are obtained from the data around M3 and M2, respectively, by applying the inversion symmetry.

Figure 1a shows the crystal structure of the new FeSe-based superconductor: (Li_1−*x*_Fe_*x*_)OHFe_1−*y*_Se (refs 36, 39). It consists of FeSe layers sandwiched in between the (Li,Fe)OH layers along the *c* direction. The in-plane lattice constant *a* or *b* is 3.78 Å, which is close to that (3.76 Å) in the bulk FeSe superconductor (*T*_c_*∼*8.5 K)^41^, but the distance of the two adjacent FeSe layers in (Li_1−*x*_Fe_*x*_)OHFe_1−*y*_Se (9.32 Å) is much larger than that in bulk FeSe (5.5 Å)^41^. This indicates a weak interaction between the two adjacent FeSe layers in (Li_1−*x*_Fe_*x*_)OHFe_1−*y*_Se and its enhanced two-dimensional nature of the electronic structure compared with bulk FeSe. In comparison, the single-layer FeSe/SrTiO_3_ film consists of a strictly two-dimensional Se-Fe-Se layer on the top of the SrTiO_3_ substrate; the thickness of the FeSe layer is 5.5 Å (Fig. 1b)^19^. The distance between the two adjacent FeSe layers in A_*x*_Fe_2−*y*_Se_2_ is 7.02 Å; however, there is an in-plane displacement between the two adjacent FeSe layers (Fig. 1c)^12^. The latest success of growing high-quality large-sized (Li_1−*x*_Fe_*x*_)OHFe_1−*y*_Se single crystals^39^ makes it possible to carry out ARPES measurements on the new superconductors. The sample we have measured in this study has a composition of (Li_0.84_Fe_0.16_)OHFe_0.98_Se (abbreviated as FeSe11111 hereafter)^39^ with a superconducting transition temperature *T*_c_*∼*41 K (Fig. 3d).

Figure 1d shows the Fermi surface mapping of FeSe11111 measured at 20 K. The spectral weight at each momentum is obtained by integrating the EDC (energy distribution curve) spectral weight within [−10,+10] meV energy window with respect to the Fermi level (*E*_F_). The measured Fermi surface contains a nearly circular electron-like Fermi surface around M points (Fig. 1d). There is no indication of Fermi crossing around the Γ (0,0) point, except that there is a very weak residual signal near the Fermi level. Such a Fermi surface topology is very similar to that found in the superconducting single-layer FeSe/SrTiO_3_ film (Fig. 1e) with a superconducting temperature at ∼55 K (ref. 20). In comparison, there are electron pockets around the Brillouin centre in the A_*x*_Fe_2−*y*_Se_2_ superconductor (Fig. 1f)^17^,^18^. The area of the electron pocket near M gives a good measure of the electron doping level for the strongly two-dimensional systems. Considering that the Fermi surface around M consists of two degenerate Fermi surface sheets, the estimated electron counting in FeSe11111 (Fig. 1d) is ∼0.08 electrons per Fe. The electron counting of ∼0.08 electrons per Fe is smaller than 0.10 electrons per Fe in single-layer FeSe film (Fig. 1e) with a *T*_c_*∼*55 K (ref. 20) and 0.12 electrons per Fe in vacuum-annealed single-layer FeSe/SrTiO_3_ film with a *T*_c_*∼*65 K (ref. 21). We note that the electron counting in both FeSe11111 and superconducting single-layer FeSe/SrTiO_3_ film is much smaller than 0.18 electrons per Fe in A_*x*_Fe_2−*y*_Se_2_ superconductor (Fig. 1f)^15^,^16^,^17^,^18^ when only considering the M point electron Fermi surface sheet.

FeSe11111 consists of FeSe layers and (Li,Fe)OH layers; therefore, the natural cleavage surface can be either FeSe surface or (Li,Fe)OH surface. By simple electron counting, both the FeSe surface and the LiOH surface are charge neutral. Substitution in the (Li,Fe)OH layers or off-stoichiometry in the FeSe layers would lead to carrier doping in the FeSe layers. In this case, no charge self-doping is expected for this sample. The composition of (Li_0.84_Fe_0.16_)OHFe_0.98_Se is obtained by structure refinement of the single-crystal X-ray diffraction data^39^. By simple electron counting, the electron doping level would be 0.12 electrons per Fe. This is slightly larger than the ∼0.08 electrons per Fe we obtained from the Fermi surface measurement. We think this difference may be caused by the composition variation among different samples^38^ or uncertainty in the composition determination. For example, neutron scattering on related sample with comparable *T*_c_ gives a composition of (Li_0.8_Fe_0.2_)OHFeSe (ref. 36). It is also possible that the electrons in the (Li,Fe)OH layers may not be fully transferred into the FeSe layers.

**Figure 2:**
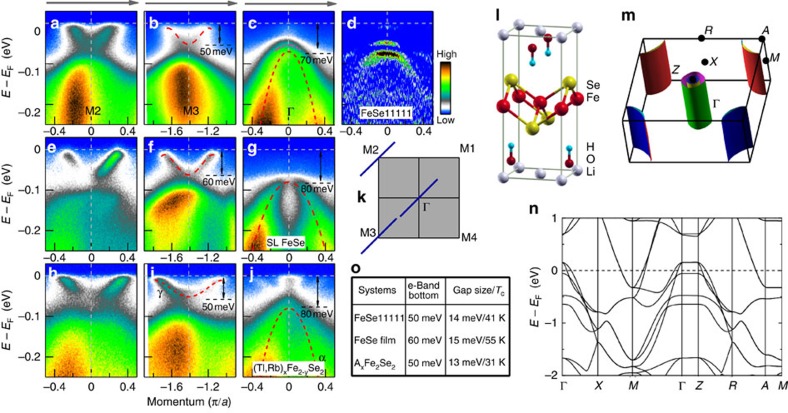
Band structure and photoemission spectra of (Li_0.84_Fe_0.16_)OHFe_0.98_Se superconductor measured along high-symmetry momentum cuts. (**a**-**c**) Photoemission images of (Li_0.84_Fe_0.16_)OHFe_0.98_Se measured at a temperature of 20 K along three high-symmetry cuts, Γ cut, M2 cut and M3 cut, respectively. (**d**) The second-derivative image of **c** with respect to the energy, to reveal the band structure better. For comparison, **e**–**j** show photoemission images of a superconducting single-layer FeSe/SrTiO_3_ film and (Tl,Rb)_*x*_Fe_2−*y*_Se_2_ measured along the same Γ cut, M2 cut and M3 cut, respectively. The location of the three momentum cuts are shown in **k**. The red dashed lines in **a**–**j** images are the guide to the eyes for the observed band structures. (**l**–**n**) DFT calculation results. (**l**) The crystal structure for the DFT calculation. (**m**) The Fermi surface and band structures along high symmetry directions are shown in **n**. In **o**, the table lists the bottom position of the electron band near M, the superconducting gap size and corresponding *T*_c_ for these three systems. The gap size for FeSe11111 will be obtained in Fig. 3e.

**Figure 3:**
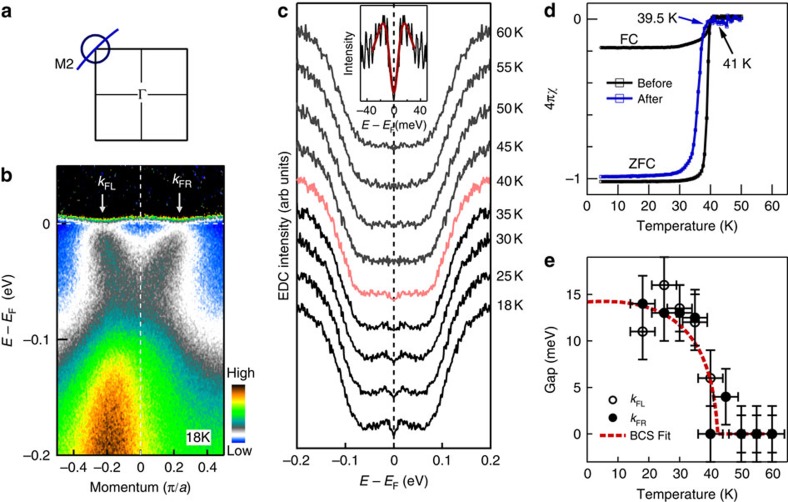
Temperature dependence of the superconducting gap of the (Li_0.84_Fe_0.16_)OHFe_0.98_Se superconductor. (**a**) Schematically, the electron-like Fermi surface near M2 point and the location of the momentum cut (blue line) across the Fermi surface. (**b**) Photoemission image measured at 18 K along the momentum cut near the M2 point (blue line in **a**). The data are divided by the corresponding Fermi distribution function, to highlight the opening of an energy gap around the Fermi level (the original data can be found in Fig. 2a). Two Fermi momenta are marked by arrows as *k*_FL_ and *k*_FR_. (**c**). Symmetrized photoemission spectra (EDCs) at the Fermi momentum *k*_FL_ measured at different temperatures. The middle-top inset shows the expanded region of the 18 K symmetrized EDC around the Fermi level and the fitting result (red line) using the formula from ref. 50 to get the gap size. (**d**) Magnetic measurement of the superconducting transition temperature (*T*_c_) for the (Li_0.84_Fe_0.16_)OHFe_0.98_Se sample we measured. Both field-cooled (FC) and zero-filed-cooled (ZFC) mode measurements show an onset *T*_c_ at ∼41 K with a sharp transition width of ∼1.5 K. (**e**) Temperature dependence of the measured superconducting gap. The gap size is obtained by picking up the peak position from the symmetrized EDCs (**c**) or by fitting the near-*E*_F_ symmetrized EDCs using the formula from ref. 50; both approaches give similar results within experimental uncertainty. The error bars are defined as s.d. The gap size from EDCs of both the left Fermi momentum (*k*_FL_) and the right Fermi momentum (*k*_FR_) are plotted. The red dashed line is a BCS gap form with a gap size of ∼14 meV at 0 temperature.

Figure 2a–c shows the band structure of FeSe11111 measured across three momentum cuts together with the density functional theory (DFT) calculated electronic structures (*l*−*n*). For comparison, the band structure measured on a superconducting single-layer FeSe/SrTiO_3_ film^20^ (Fig. 3e–g) and on (Tl,Rb)_*x*_Fe_2−*y*_Se_2_ superconductor^17^ (Fig. 3h-j) along the same three momentum cuts are also presented. Overall, the band structure of FeSe11111 is very similar to that of the superconducting single-layer FeSe/SrTiO_3_ film with slight quantitative difference in the band position. Near the Γ point, two hole-like bands can be resolved for FeSe11111 (Fig. 2c) that are well below the Fermi level, as seen more clearly in the corresponding second-derivative image (Fig. 2d). We note that there is a very weak signal near the Fermi level at Γ point, which may be due to the leakage from a band above the Fermi level. Similar multiple hole-like bands have also been observed near Γ in the superconducting single-layer FeSe/SrTiO_3_ films^21^ and in K_*x*_Fe_2−*y*_Se_2_ superconductor^18^, with slight variation in the band top position. In comparison, the electronic structure of (Tl,Rb)_*x*_Fe_2−*y*_Se_2_ superconductor shows two electron-like bands crossing the Fermi level (Fig. 2j)^17^. Around M2 and M3 points, we observed in FeSe11111 parabolic electron-like bands crossing the Fermi level (Fig. 2a,b), forming the electron pocket around M. These are very similar to those observed in the superconducting single-layer FeSe/SrTiO_3_ film (Fig. 2e,f). Careful examination indicates that the Fermi momentum (*k*_F_) for the electron pocket of FeSe11111 is ∼0.22 *π*/*a* (lattice constant *a*=3.78 Å) (Fig. 2a,b), which is slightly smaller than 0.25 *π*/*a* in the superconducting single-layer FeSe/SrTiO_3_ film (Fig. 2e,f)^20^, both being significantly smaller than 0.35 *π*/*a* in (Tl,Rb)_*x*_Fe_2−*y*_Se_2_ superconductor (Fig. 2h,i)^17^. As a summary in Fig. 2o, the bottom of the electron-like band in FeSe11111 is ∼50 meV below the Fermi level(Fig. 2b), slightly shallower than the 60 meV in the superconducting single-layer FeSe/SrTiO_3_ film (Fig. 2f)^20^ but similar to the ∼50 meV in the (Tl,Rb)_*x*_Fe_2−*y*_Se_2_ superconductor (Fig. 2i)^17^. The combined information of the Fermi momentum and the band width gives a good measure of the electron effective mass that is 2.9, 2.7 and 6.1 *m*_*e*_ (*m*_*e*_ is free electron mass) for FeSe11111, superconducting single-layer FeSe/SrTiO_3_ film and the (Tl,Rb)_*x*_Fe_2−*y*_Se_2_ superconductor, respectively^17^,^20^. It is interesting to note that electron effective mass for the electron-like band near *M* is similar for FeSe11111 and superconducting single-layer FeSe/SrTiO_3_ film, but both are much smaller than that in (Tl,Rb)_*x*_Fe_2−*y*_Se_2_ superconductor^17^.

To further understand the measured electronic structure, the first-principles electronic structure calculations were carried out with the projector augmented wave method^42^,^43^ as implemented in the VASP software^44^,^45^,^46^ with a electron doping 0.1*e* per Fe in the non-magnetic state, which is close to our sample state. The crystal structure (Fig. 2l) and lattice constant are from ref. 36. Overall, the calculated Fermi surface (Fig. 2m) shows similar result with that of the bulk FeSe (ref. 47) but with enhanced two-dimensional characteristics, consistent with the larger spacing between the two adjacent FeSe layers in (Li,Fe)OHFeSe. Extra bands around −2.5 and −1.0 eV are present that are from (Li,Fe)OH layers, which are too deep to give contribution around the Fermi level. It is apparent that there is an obvious discrepancy between the band structure calculations and our measurements. For example, the hole pockets near Γ point in the calculations are absent in the measured results. This situation is similar to that of the single-layer FeSe/SrTiO_3_ films and other FeSe-based materials^23^. More theoretical efforts are needed to understand the experimental results.

The natural cleavage surface of FeSe11111 consists of both the FeSe surface and the (Li,Fe)OH surface; therefore, our ARPES signal should come from both surfaces. We believe our ARPES signal is dominated by the FeSe layers because of the following reasons: (1) our density functional theory band structure calculations (Fig. 2) indicate that the bands from (Li,Fe)OH layers lie ∼2.5–1.0 eV below the Fermi level. Thus, the low-energy electronic states near the Fermi level are mainly from the FeSe layers. This is consistent with the electronic structure similarity between (Li,Fe)OHFeSe and the single-layer FeSe/SrTiO_3_ that has no (Li,Fe)OH layers. (2) Recent scanning tunneling microscope (STM) study on cleaved (Li,Fe)OHFeSe also indicated that the low-energy signal is dominated by the FeSe cleavage surface^48^,^49^. We note that STM measurement on the (Li,Fe)OH layer reveals that there is a tiny electron pocket around Γ (ref. 49). As we mentioned above, we also observe a very weak signal near the Fermi level at Γ point (Figs 1d and 2c). Whether this weak signal near Γ is due to the tiny electron pocket identified from STM^49^ needs further investigation.

### Superconducting gap

The clear identification of electron-like Fermi surface sheet near M point makes it possible to investigate the superconducting gap in this new FeSe11111 superconductor. We start by examining the temperature dependence of the superconducting gap. Figure 3b shows the original photoemission image measured at a low temperature of 18 K along the momentum cut (its location is shown in Fig. 3a) crossing the electron-like Fermi surface around M2. The data are divided by the corresponding Fermi distribution function; the spectral weight suppression near the Fermi level indicates the opening of the superconducting gap (Fig. 3b). Following the procedure commonly used in the study of high-temperature cuprate superconductors^50^, the symmetrized photoemission spectra (EDCs) measured on one Fermi momentum at different temperatures are shown in Fig. 3c. There is a clear gap opening at low temperatures, as indicated by a dip at the Fermi level in the symmetrized EDCs (Fig. 3c). With increasing temperature, the dip at *E*_F_ is gradually filled up and becomes almost invisible above 40 K. We used two methods to extract the gap size in the superconducting state. The first method is to pick the peak position in the symmetrized EDCs, while the second one is to fit the symmetrized EDCs with the phenomenological formula proposed by Norman *et al.*^50^. The top-middle inset of Fig. 3c shows the symmetrized EDC at 18 K and the fitting result. Both methods give consistent gap size within an experimental uncertainty of 2 meV. Figure 3e shows the measured gap size at different temperatures from symmetrized EDCs for both Fermi momenta. The gap size basically follows a Bardeen-Cooper-Schrieffer form with a *T*_c_ around (42±2) K and a gap size of ∼14 meV at 0 temperature (Fig. 3e). This is in excellent agreement with the *T*_c_*∼*41 K from direct magnetic measurement (Fig. 3d). We note that FeSe11111 is very sensitive to air and processing temperature. To make sure there is no change on the superconductivity of our sample during the ARPES measurements, we carried out magnetic measurements on the measured sample before and after the ARPES experiment. Figure 3d shows the magnetic measurement before ARPES measurement; both the field-cooled and zero-field-cooled modes give similar magnetic onset *T*_c_*∼*41 K with a sharp transition of ∼1.5 K. The sharp transition and its 100% diamagnetic shielding demonstrate the high quality of the measured single crystal. After ARPES experiment (Fig. 3d), the magnetic measurement on the cleaved sample shows an onset *T*_c_*∼*40 K with a sharp transition of ∼1.5 K. These indicate that there is negligible superconductivity degradation on the sample during the sample handling and ARPES measurements.

**Figure 4:**
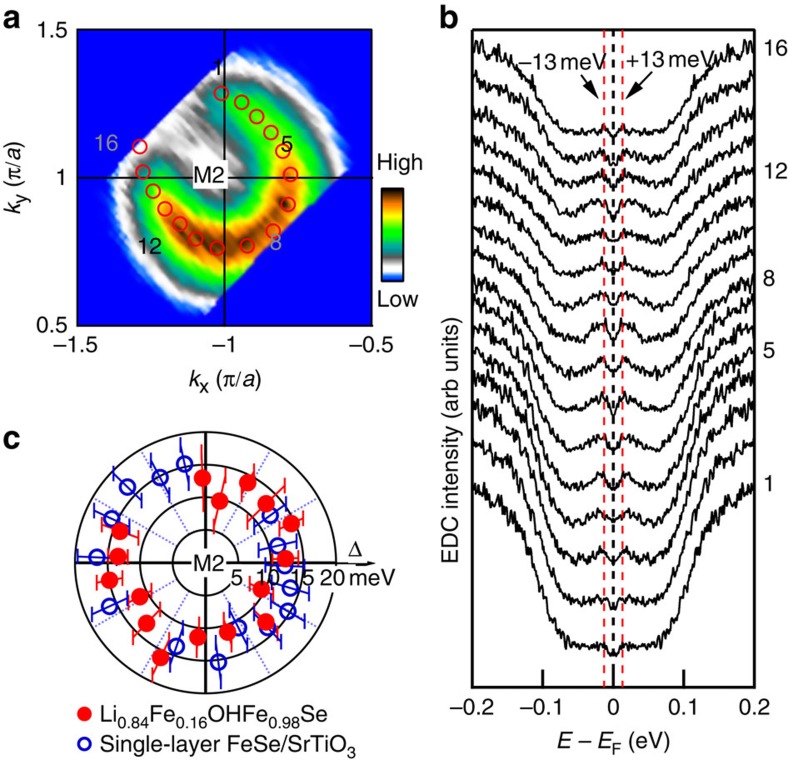
Momentum dependence of the superconducting gap of (Li_0.84_Fe_0.16_)OHFe_0.98_Se superconductor. (**a**) Fermi surface mapping near M2 point. The corresponding Fermi crossings are marked by red empty circles with numbers from 1 to 16 (for clarity, the intermittent points are not labelled). (**b**) Symmetrized EDCs measured at 20 K from Fermi crossing 1 to 16 along the measured electron-like Fermi surface around M2. The red dashed lines represent the energy positions of ±13 meV with respect to the Fermi level. (**c**) Momentum dependence of the superconducting gap (red solid circles) along the Fermi surface around M2 for (Li_0.84_Fe_0.16_)OHFe_0.98_Se superconductor. For comparison, the gap along the electron-like Fermi surface around M2 of the superconducting single-layer FeSe film is also plotted (blue empty circles)^20^. The error bars are defined as s.d.

Now we come to the momentum dependence of the superconducting gap in the FeSe11111 superconductor. For this purpose, we took high-resolution Fermi surface mapping (energy resolution of 4 meV) of the electron pocket around M2 at 20 K in the superconducting state (Fig. 4a). The corresponding Fermi momenta are identified, marked by red empty circles and labelled by numbers from 1 to 16 in Fig. 4a. Figure 4b shows symmetrized photoemission spectra (EDCs) at the Fermi momenta around the Fermi surface. The extracted superconducting gap is plotted in Fig. 4c. The superconducting gap is nearly isotropic with a gap size at (13±2) meV; there is no gap node (zero gap) around the Fermi surface. For comparison, the gap size around the electron-like Fermi surface at M2 point for the superconducting single-layer FeSe/SrTiO_3_ film^20^ is also plotted (Fig. 4c). It is also nearly isotropic without gap node, although its magnitude (∼15 meV) is slightly larger than that in FeSe11111. In FeSe11111, the bonding between the FeSe layer and the adjacent (Li,Fe)OH layer is rather weak (van der Waals bonding). The separation between the two adjacent FeSe layers is large and their mutual interaction is expected to be rather weak. These make its behaviours close to the single-layer FeSe film that shows strong two dimensionality. It is thus reasonable to conclude that there is no nodes of the superconducting gap in the FeSe11111 superconductor.

## Discussion

From the above results, we have found that the FeSe11111 superconductor exhibits similar behaviours to the superconducting single-layer FeSe/SrTiO_3_ film, in terms of the Fermi surface topology, band structure and the superconducting gap symmetry. Among all the iron-based superconductors discovered so far, the majority of them show common electronic structure with hole-like Fermi surface sheets around the Γ point and electron-like Fermi surface near the M point^1^. The A_*x*_Fe_2−*y*_Se_2_ superconductor^3^,^14^,^15^,^16^,^17^,^18^ and the superconducting single-layer FeSe/SrTiO_3_ films^20^,^21^,^22^,^23^ have been the only two exceptions that have distinct Fermi surface topology, that is, no hole-like Fermi surface at the Γ point and there are only electron pockets. The present work has established the FeSe11111 superconductor as a new third exception on the list. It has been proposed that the electron scattering between the hole-like bands near Γ and electron-like bands near M is the cause of electron pairing in the iron-based superconductors^5^,^6^,^7^,^8^,^9^,^10^,^11^. The discovery of only electron-like Fermi surface in the A_*x*_Fe_2−*y*_Se_2_ superconductor and the superconducting single-layer FeSe/SrTiO_3_ films challenges such a Fermi surface nesting scenario. However, some material-specific properties prevent us from making a decisive conclusion. The A_*x*_Fe_2−*y*_Se_2_ superconductor is well known for its phase separation problem; the superconducting phase occupies only a small fraction of the entire sample volume and the nature of the superconducting phase remains unclear^3^,^14^,^24^,^25^,^26^,^27^. The coexistence of the superconducting phase and the insulating 245 phase makes people wonder whether the interface between them is responsible for the observed superconductivity. On the other hand, the surprising discovery of high-temperature superconductivity in the single-layer FeSe/SrTiO_3_ films indicates that interface may play a significant role in inducing or enhancing superconductivity^19^,^20^,^21^,^22^,^23^,^28^,^29^,^30^,^31^,^32^,^33^,^34^. In this sense, the complications of phase separation and interface effect hinder the direct comparison of the A_*x*_Fe_2−*y*_Se_2_ superconductors and superconducting single-layer FeSe/SrTiO_3_ films with most other iron-based superconductors. The newly discovered FeSe11111 superconductor is advantageous in that it is a single-phase bulk superconductor, free from complications of phase separation and interface effect, thus making it possible to compare directly with other iron-based bulk superconductors. The observation of high-temperature superconductivity with a *T*_c_ at 41 K in bulk FeSe11111 superconductor that has electron-pocket-only Fermi surface topology makes the strongest and most decisive case that the hole-like Fermi surface around Γ is not necessary for the superconductivity in the iron-based superconductors. This rules out Fermi surface nesting scenario as the common pairing mechanism for the iron-based superconductors and points to the significant role of the electron pockets near M in governing high-temperature superconductivity in the iron-based superconductors.

The present work also has important implications on the high-temperature superconductivity in the single-layer FeSe/SrTiO_3_ films. First, our results indicate that the electron-pocket-near-M-only Fermi surface topology is not confined to the superconducting single-layer FeSe/SrTiO_3_ films only; instead, it can also be present in bulk superconductors. Our experimental results have shown that this form of Fermi surface topology is favourable for achieving high-temperature superconductivity in the iron-based superconductors. Second, it is remarkable that *T*_c_ as high as 41 K can be achieved in the bulk FeSe11111 superconductor where there is no strain effect, no interface effect and no strong electron–phonon coupling effect^29^ involved as in the superconducting single-layer FeSe/SrTiO_3_ films. It is known from the superconducting single-layer FeSe/SrTiO_3_ films that *T*_c_ can be tuned by varying the electron doping level^21^. Therefore, it is possible that *T*_c_=41 K is lower in FeSe11111 than that in the superconducting single-layer FeSe films (∼65 K or higher), because the electron doping level in FeSe11111 is low. This raises a big possibility that *T*_c_ of the FeSe11111 superconductor may be further increased if higher electron doping is made possible and the superconductor–insulator transition^40^ can be avoided. The high *T*_c_ and its possible enhancement with increasing doping in the FeSe11111 superconductor provide key insight on understanding the superconductivity in the single-layer FeSe/SrTiO_3_ films. Third, we note that the in-plane lattice constant a or b of the FeSe11111 superconductor (3.78 Å)^36^,^39^ is comparable to the primary bulk FeSe superconductor (3.76 Å), but significantly smaller than the single-layer FeSe film epitaxially grown on the SrTiO_3_ substrate (3.905 Å). Considering high *T*_c_=41 K and its possible enhancement on further elecron doping in the FeSe11111 superconductor, this indicates that the interface strain does not play primary role in determining *T*_c_. On the other hand, the distance between the two adjacent FeSe layers seems to have strong correlation with superconductivity in the FeSe-based superconductors. As the distance changes from 5.5 Å for bulk FeSe to 9.32 Å in the FeSe11111 superconductor, and to infinity in the single-layer FeSe/SrTiO_3_ films, T_c_ increases from 8.5 K to 41 K, to 65 K or higher (Fig. 1). In the meantime, the top of the hole-like band at Γ changes its position from above the Fermi level for bulk FeSe (ref. 51) to 50∼70 meV below the Fermi level for FeSe11111, to 80 meV below the Fermi level for the superconducting single-layer FeSe films (Fig. 2). These results again indicate that suppression of the hole-like bands near the Γ point is favourable for achieving high-temperature superconductivity in the iron-based superconductors.

In summary, by performing high-resolution ARPES measurements on the FeSe-based superconductor, FeSe11111, we have discovered that it exhibits strikingly similar electronic behaviours to the superconducting single-layer FeSe/SrTiO_3_ film, in terms of the Fermi surface topology, band structure and the superconducting gap symmetry. This is the first single-phase bulk superconductor with a relatively high *T*_c_*∼*41 K that shows electron-like Fermi-pockets-only Fermi surface topology that offers a direct comparison with other iron-based bulk superconductors.

## Methods

### Single crystal preparation

High-quality (Li_1−*x*_Fe_*x*_)OHFe_1−*y*_Se single crystals were grown by hydrothermal approaches^39^. Large crystals of K_0.8_Fe_1.6_Se_2_ (nominal 245 phase) are specially grown and used as a kind of matrix for a hydrothermal ionic exchange reaction. The K ions in K_0.8_Fe_1.6_Se_2_ are completely released into solution after the hydrothermal reaction process. Single-crystal X-ray diffraction (XRD) and powder XRD measurements confirm the high quality and high purity of the FeSe11111 single crystals. In particular, no trace of K ions in the FeSe11111 crystals is detected by energy dispersive X-ray spectrometry and Inductively-coupled plasma atomic emission spectroscopy (ICP-AES) analyses, confirming a complete release of K ions after the hydrothermal ionic exchange. The sample is sensitive to air and treatment temperature due to the existence of hydroxide. All the samples for ARPES measurements were prepared in glove box and vacuum case within a short time and then transferred to ARPES measurement chamber to be kept at low temperature during the ARPES experiments.

### High-resolution ARPES measurements

High-resolution ARPES measurements were carried out on our lab system equipped with a Scienta R8000 electron energy analyser^52^. We use helium discharge lamp as the light source, which can provide photon energies of *hυ*=21.218 eV (helium I). The energy resolution was set at 4 meV for both the Fermi surface mapping and band structure measurements (Figs 1 and 2) and for the superconducting gap measurements (Figs 3 and 4). The angular resolution is ∼0.3°. The Fermi level is referenced by measuring on a clean polycrystalline gold that is electrically connected to the sample. The sample was cleaved and measured in vacuum with a base pressure better than 5 × 10^−11^ Torr.

## Additional information

**How to cite this article:** Zhao, L. *et al.* Common electronic origin of superconductivity in (Li,Fe)OHFeSe bulk superconductor and single-layer FeSe/SrTiO_3_ films. *Nat. Commun.* 7:10608 doi: 10.1038/ncomms10608 (2016).
